# Optimizing Tinnitus Management: The Important Role of Hearing Aids with Sound Generators

**DOI:** 10.3390/audiolres14040057

**Published:** 2024-08-06

**Authors:** Yuki Kosugi, Toru Miwa, Yuka Haruta, Kosuke Hashimoto, Shoko Kato

**Affiliations:** 1 Department of Otolaryngology, Osaka Metropolitan University, 1-4-3 Asahi-machi, Abeno-ku, Osaka 545-8585, Japan; yukikosugi@outlook.jp (Y.K.); yuka.nao.saki@gmail.com (Y.H.); hashimoto@ent-ocu.com (K.H.); shoko.kato@omu.ac.jp (S.K.); 2 Department of Otolaryngology-Head and Neck Surgery, Graduate of School of Medicine, Kyoto University, 54 Shogoin Kawahara-cho, Sakyo-ku, Kyoto 606-8507, Japan

**Keywords:** tinnitus, hearing aid, tinnitus retraining therapy, sound generators, long-term result

## Abstract

Hearing aids (HAs), especially those with sound generators (SGs), are used in the management of tinnitus. However, their comparative efficacies and long-term outcomes remain unknown. Therefore, we investigated the efficacy and long-term outcomes of tinnitus therapy using various HA SG models. We retrospectively reviewed 666 patients with chronic tinnitus characterized by persistent symptoms for >6 months. At the initial visit, the patients received educational counselling on tinnitus (Utsunomiya method) and completed a comprehensive questionnaire comprising the tinnitus handicap inventory, a visual analog scale, the state–trait anxiety inventory, and the emotional intelligence scale. The scores were compared among various models of HA SGs and SGs. The patients underwent follow-ups for up to 2 years. Our results indicated that tinnitus retraining therapy using SGs and conventional HAs effectively managed chronic tinnitus. The prolonged use of HAs appeared to exacerbate tinnitus symptoms, emphasizing the superior long-term effectiveness of SG HAs, particularly ZEN (Widex ZEN, WS Audiology, Lynge, Denmark). Our findings indicate that HAs are useful in the first year, but their prolonged use may exacerbate tinnitus symptoms, whereas HA SGs are effective in the long term. Future studies should account for the variations in tinnitus treatment effects based on the type of sound employed.

## 1. Introduction

Tinnitus affects 10–15% of the global population; clinically problematic cases constitute approximately 20% of all cases, which is equivalent to 2–3% of the world population [1]. In Japan, an estimated 3 million individuals experience bothersome tinnitus that significantly affects their daily lives [2]. Tinnitus retraining therapy (TRT), encompassing acoustic therapy and educational counselling, is a viable treatment option. However, the selection of specific acoustic therapies depends on the presence or absence of subjective hearing loss and the extent to which tinnitus disrupts daily life [3]. The overarching objective of acoustic therapy in TRT is twofold: to diminish the intensity of tinnitus perception and to foster adaptation to the condition of tinnitus. The various acoustic modalities used in TRT include environmental music, sound generators (SGs), and hearing aids (HAs) [4]. Although HAs were initially designed to augment auditory function, they have recently demonstrated efficacy in tinnitus management [5,6,7]. Accordingly, acoustic therapy involving HAs was strongly recommended (1A) in the 2019 edition of the Guidelines for the Treatment of Tinnitus [2]. The tinnitus practice guidelines from the American Academy of Otolaryngology—Head and Neck Surgery [8] and the German guidelines [9] also support this approach. Moreover, more than 80% of the individuals with tinnitus exhibit concurrent hearing loss [10], underscoring the increasing importance of HAs in the comprehensive management of tinnitus.

The rationale for employing acoustic therapy with HAs in tinnitus treatment is grounded in the “neurophysiological model of tinnitus” proposed by Jastreboff [11]. According to this model, the initiation of tinnitus stems from a decline in sound input at the periphery coupled with increased activity in the auditory center. The “tinnitus distress model” posits the existence of a detrimental cycle triggered by the synchronization of neural activity with non-auditory areas, constituting the distress network. This network encompasses a heightened attention, the perception of danger, anxiety, depression, and an association with negative memories such as “feelings” or “thoughts” (non-existing memories) [11]. In the last 35 years, various conceptional models of tinnitus have been proposed. Zenner and Zalaman’s cognitive desensitized therapy reduces the tinnitus burden and improves patients’ quality of life [12]. Zenner et al. argued that tinnitus sensitization arises from interpreting sound as unpredictable and fear-inducing, leading to helplessness [13]. Conversely, McKenna et al.’s cognitive–behavioral model links distress to negative thoughts about tinnitus, provoking emotional distress and maladaptive behaviors [14], whereas Ghodratitoostani’s neurofunctional tinnitus model (NfTM) emphasizes the role of cognitive–emotional appraisal processes (CAAPs) in tinnitus distress, proposing stages of neutral and clinical distress [15]. Furthermore, a new conceptual cognitive framework (CCF) incorporates these cognitive processes to model and test causality in tinnitus distress and management [16].

HAs are pivotal in tinnitus management, and are primarily used to mitigate central nervous system overactivity by compensating for diminished auditory inputs. Additionally, they reduce the tinnitus contrast by supplying ample sound and alleviating stress by facilitating communication. The application of HAs in tinnitus therapy is deemed an effective intervention that targets both the auditory and non-auditory domains, particularly within the framework of the mechanism governing tinnitus exacerbation [17]. Consequently, the functions of HAs in TRT are multifaceted; they aim to alleviate brain excitation, divert attention from tinnitus, and provide relief in sonically enriched environments. Previous studies have also demonstrated empirical evidence supporting the efficacy of HAs in this regard [5,6,7].

Recently, companies manufacturing HAs have introduced new models, hereafter referred to as SG HAs, in addition to conventional models. SG HAs comprise a masker or an SG that serves as a partial masker for adaptation to tinnitus, in addition to the HA [18]. Historically, therapeutic sounds compatible with HAs were limited to uniform audible noises. However, recent advancements have expanded the repertoire to include fluctuating sounds, natural and environmental sounds, and music composed within the HA using fractal theory (Zentone^®^, Widex, Lynge, Denmark) [19,20]. Makitie (Tokyo, Japan) provides four types of noises, including white, pink, and high- and low-frequency-enhanced, whereas Sivantos (Singapore) offers five types of noises, including white, speech (low-frequency-enhanced), pink, high-frequency, and fluctuation. The latest model by Sivantos introduced four new wave sounds, adding to the diversity of available therapeutic options.

Henry et al. conducted a randomized controlled trial and reported that both traditional and SG HAs were effective for tinnitus with no statistically significant differences [17]. In contrast, Jalilvand et al. reported that SGs alone were less effective compared to HAs and SG HAs in 974 patients with tinnitus caused by acoustic trauma [21]. Consequently, the comparative efficacies and long-term outcomes of SG HAs, conventional HAs, and stand-alone SG devices remain insufficiently documented and require further investigation.

Therefore, the present study aimed to examine the efficacy and long-term outcomes associated with each model to provide better tinnitus treatment for patients with chronic tinnitus.

## 2. Materials and Methods

### 2.1. Patients

Among the patients with tinnitus who visited our department between January 2012 and December 2020, we retrospectively reviewed 666 patients with chronic tinnitus characterized by persistent symptoms for over 6 months. The inclusion criteria were an age ≥ 18 years and a minimum follow-up period of 2 years. Individuals < 18 years old and those with missing initial data were excluded. This study was approved by our institutional review board (IRB number: 2020-165). Written informed consent was obtained for this study. This study adhered to the principles outlined in the Declaration of Helsinki.

### 2.2. Study Design

At the initial visit, educational counselling on tinnitus (Utsunomiya method) [5] was administered, and the patients were asked to complete a comprehensive questionnaire. The questionnaire included the tinnitus handicap inventory (THI) [22], which assessed the extent of distress and disruption in daily life owing to tinnitus; a visual analog scale (VAS) that gauged tinnitus-related distress; the state–trait anxiety inventory (STAI) [23]; and the emotional intelligence scale (EQS), which is designed to evaluate emotional intelligence [24,25]. The STAI, which measures anxiety, comprises 40 questions scored on a scale of 1–4 points, categorized into state and trait anxieties [23]. The EQS is structured into three domains: (1) self, (2) interpersonal, and (3) situational responses, each comprising three corresponding factors. The nine response factors are self-insight, self-motivation, self-control, empathy, altruism, interpersonal control, situational insight, leadership, and situational control, with a total of 21 sub-factors. Each sub-factor comprises three questions, totaling sixty-five questions, including two response reliability criteria rated on a scale of 0–4. The questionnaire administration required 10 min, whereas scoring required 15 min, with higher scores indicating a greater emotional intelligence [24,25].

Patients with subjective hearing loss were fitted with HAs or SG HAs, whereas those without hearing loss received SGs. The TRT, including the Utsunomiya method’s educational counselling, was initiated after HA, SG HA, or SG fitting. After the initial visit, the patients were scheduled for follow-ups at 1, 3, and 6 months and 1, 1.5, and 2 years, during which the Utsunomiya method’s educational counselling on tinnitus was provided. Six sessions were performed, with each lasting approximately 30 min. At the 1- and 2-year follow-ups, the patients reported their scores on the THI, VAS, STAI, and EQS. The HA group used a combination of devices from Widex Co., Ltd. (Lynge, Denmark), Makitie Co., Ltd. (Tokyo, Japan), and Sivantos Pte. Ltd. (Singapore). The SG HA group was divided into ZEN and TTHA/TTC groups based on devices from Widex and Makitie, respectively. The SG-alone group was further divided into TTSG/TT and TCI groups based on devices from Makitie and Sivantos, respectively. Additionally, the control group (CNT) included patients who did not use HAs, SG HAs, or SGs (Table 1). The individuals in the control group received only educational counselling (Utsunomiya method). Educational counseling for tinnitus differs from the general concept of counseling in that it is educational or explanatory counseling (provision of information) for tinnitus. The patient is informed about the mechanism of hearing, the presence or absence of organic disease, the mechanism of tinnitus, the mechanism of tinnitus exacerbation, the treatment options, the treatment goals, and the general progression.

We evaluated the rate of improvement in tinnitus by defining “tinnitus improvement” as patients whose THI score decreased by more than 20 points from the first THI score or by at least 16 points at the end of the assessment. We calculated the number of patients with “tinnitus improvement” in each group. The improvement rate was calculated by dividing the score at the end of the evaluation by the score at the initial visit to evaluate changes in the VAS, STAI, and EQS. The primary outcome measure was the rate of tinnitus improvement, and the secondary outcomes were changes in the VAS, STAI, and EQS scores.

### 2.3. Statistical Analyses

The age, VAS, STAI, and EQS were analyzed using a one-way analysis of variance (ANOVA), and post hoc analyses were performed using Tukey’s method. The sex distribution was assessed using Fisher’s exact test. Missing values are visually represented using forest plots. The statistical analyses were performed using GraphPad Prism, version 9.5.0 (Windows GraphPad Software, San Diego, CA, USA). The statistical significance was set at *p* < 0.05.

## 3. Results

### 3.1. Demographic Information

Of the 666 patients, 517 were accessible for a 2-year follow-up. The distribution of patients in each group, the mean age, and the sex ratios are presented in Table 1. No statistically significant differences were observed in the age or sex ratios across the groups.

### 3.2. Improvement in Tinnitus

#### 3.2.1. Group-Wise Comparisons among the CNT, HA, SG HA, and SG Groups

At 1 year, the HA, SG HA, and SG groups demonstrated greater rates of improvement in their THI scores than the CNT group [HA: 53.6% (*n* = 28), SG HA: 43.9% (*n* = 271), and SG: 41.9% (*n* = 74) vs. CNT: 20.8% (*n* = 53); Figure S1]. Similarly, after 2 years of follow-up, the SG HA and SG groups demonstrated greater rates of improvement in their THI scores than the CNT group [SG HA: 50.9% (*n* = 271) and SG: 37.8% (*n* = 74); Figure S1]. However, the HA group demonstrated lower rates of improvement than the CNT group (HA: 10.7% (*n* = 28) vs. CNT: 17.0% (*n* = 53); Figure S1).

#### 3.2.2. Group-Wise Comparisons among the CNT, HA, Various Models of SG HA (ZEN and TTHA/TTC), and SG (TT/TTSG and TCI) Groups

**Figure 1:**
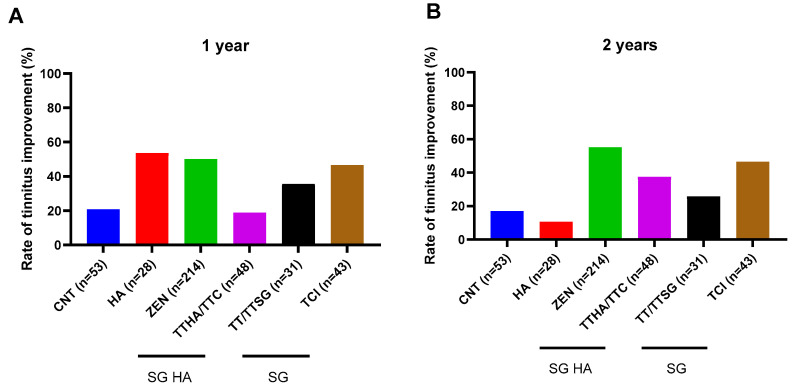
Comparison of improvement ratios for tinnitus handicap inventory (THI) among devices. (**A**) 1 year; (**B**) 2 years.

The tinnitus improvement rates are shown in Figure 1. Group-wise comparisons of the THI improvement rate revealed that, at 1 year, the ZEN group (50.0%; SG HAs) demonstrated greater improvement rates than the TCI (46.5%), TT/TTSG (35.5%; SG), and TTHA/TTC (18.8%; SG HAs) groups (Figure 1). Similarly, after 2 years of follow-up, the ZEN group (55.1%; SG HAs) demonstrated better rates of improvement than the TTHA/TTC (37.5%; SG HAs), TCI (46.5%), and TT/TTSG (25.8%; SG) groups (Figure 1). The means ± standard deviations of the THI scores are shown in Table 2.

### 3.3. Improvement in VAS Score

**Figure 2:**
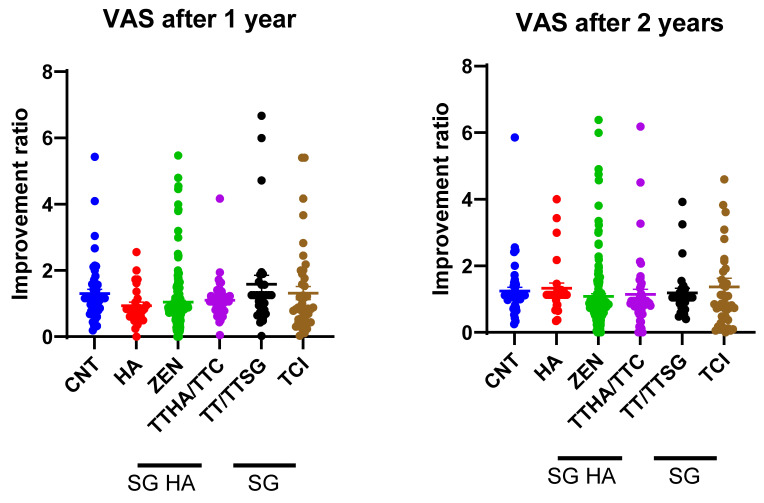
Comparison of improvement ratio for visual analog scale (VAS) among devices.

The VAS scores are presented in Figure 2. Notably, at the 1-year follow-up, the ZEN group exhibited significantly greater improvement than the TT/TTSG groups (−0.53 [−1.06 to −0.01], *p* = 0.04). However, no statistically significant differences were observed among the groups after 2 years of follow-up.

### 3.4. STAI Improvement Rate

**Figure 3:**
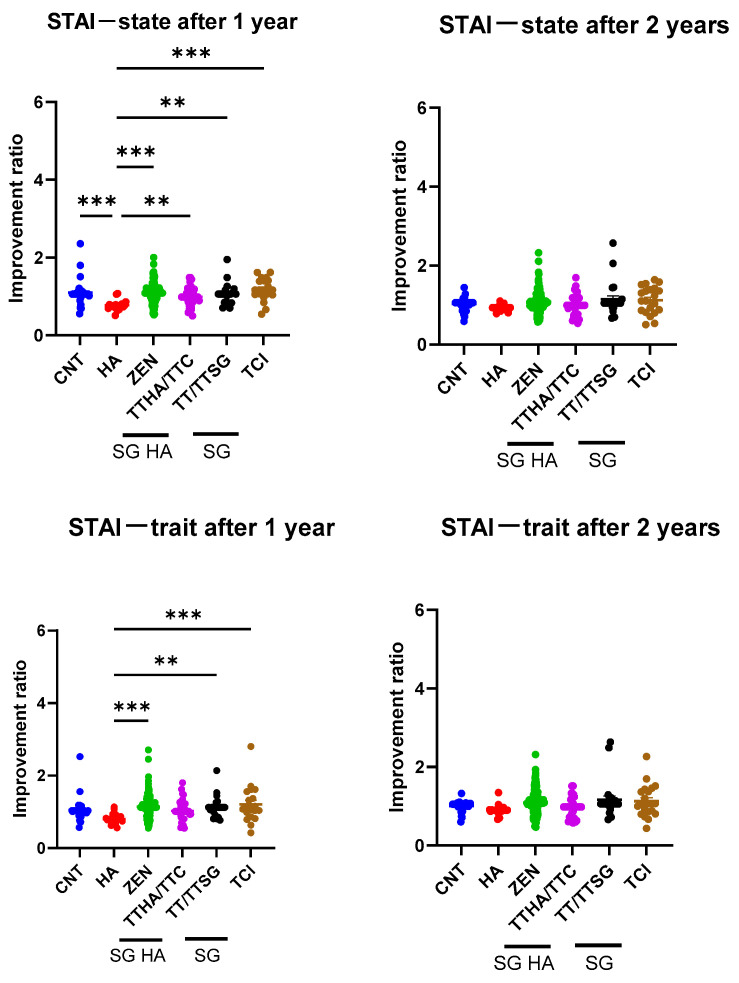
Comparison of improvement ratio for state–trait anxiety inventory (STAI) among devices. ** *p* < 0.01, *** *p* < 0.001. The two panels on the left show the STAI—state, and the two on the right show the STAI—trait.

Figure 3 presents the STAI scores, with lower values indicating a higher rate of improvement. The HA and TTHA/TTC groups exhibited significant improvements in their STAI—state after 1 year of follow-up. Inter-group comparisons showed that the HA group demonstrated significantly greater improvement in the STAI—state than the CNT, ZEN, TTHA/TTC, TT/TTSG, and TCI groups (CNT: 0.32 [0.13–0.51], *p* < 0.001; ZEN: −0.32 [−0.48 to −0.16], *p* < 0.001; TTHA/TTC: −0.22 [−0.41 to −0.03], *p* = 0.008; TT/TTSG: −0.29 [−0.51 to −0.08], *p* < 0.001; and TCI: −0.41 [−0.62 to −0.20], *p* < 0.001). However, no significant differences were observed among the models and CNT at 2 years.

At the 1-year follow-up, only the HA group showed an improvement in the STAI—trait (Table 2). The between-group comparisons showed that the HA group demonstrated a significantly greater improvement in the STAI—trait than the CNT, ZEN, TT/TTSG, and TCI groups (CNT: 0.25 [0.01–0.48], *p* = 0.03; ZEN: −0.34 [−0.54 to −0.14], *p* < 0.001; TT/TTSG: −0.33 [−0.60 to −0.07], *p* = 0.005; and TCI: −0.40 [−0.67 to −0.13]; *p* < 0.001). However, no significant differences were seen among the groups at 2 years.

### 3.5. Improvement in EQS Scores

**Figure 4:**
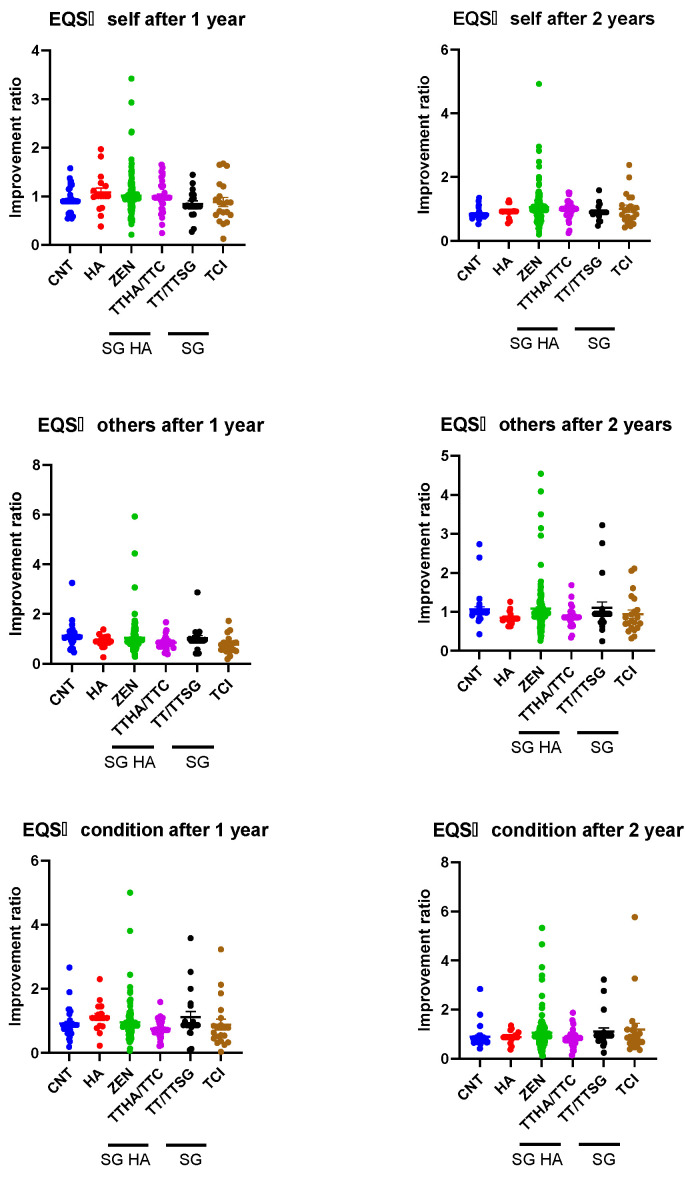
Comparison of improvement ratio for emotional intelligence scale (EQS) among devices. The upper panels present the EQS—self, the middle panels present the EQS—others, and the lower panels present the EQS—conditions.

Figure 4 presents the EQS scores, with lower values indicating a higher rate of improvement. No differences were observed in the between-group comparisons in EQS among the groups for the self, others, or condition categories.

## 4. Discussion

Our present study showed that HAs, SG HAs (especially ZEN), and SGs exhibited a superior performance in tinnitus improvement than the CNT at the 1-year follow-up. Additionally, SG HAs and SGs exhibited a superior performance in tinnitus improvement compared to the CNT at the 2-year follow-up, while HAs exhibited an inferior performance. Almost no significant differences were noted in the VAS and EQS scores among the models and the CNT. Furthermore, we found an amelioration in the STAI—state and STAI—trait scores using HAs at the 1-year follow-up; however, there were no significant differences among the model and CNT groups at the 2-year follow-up.

Previous findings on HAs for tinnitus have suggested that HAs for hearing loss improve tinnitus by modulating the auditory input from the periphery to the thalamus in an ascending fashion, thereby controlling the auditory thalamocortical firing rhythm modulation [10,17]. In addition to this model, alternative explanations for tinnitus include the stochastic resonance model by Krauss et al., 2016, which emphasizes the role of SR in enhancing weak auditory signals [26]. Furthermore, various central gain approaches suggest that an increased central neuronal gain compensates for a reduced auditory input, leading to hyperactivity and tinnitus [27]. Collectively, these alternative models offer valuable perspectives on the mechanisms underlying tinnitus and potential avenues for tinnitus reduction. Thus, our finding that HAs improved tinnitus distress at the 1-year follow-up is consistent with that of previous studies [4,5,10,17,18]. Notably, in the second year of treatment, tinnitus improved in the SG HA and SG groups, similar to the results at the 1-year follow-up. However, the rate of tinnitus improvement in patients treated only with HAs tended to worsen in the second year compared to that in the first year, suggesting that noise, music, and other acoustics may have had an additional therapeutic effect. In other words, the effective control of tinnitus can be achieved if SG HAs and HAs are introduced in the early stages of tinnitus treatment to decrease the degree of distress, followed by continued educational counselling and the introduction of acoustic therapy.

We found that ZEN had a positive effect on the tinnitus distress levels. ZEN was the only SG HA in this study that incorporated music, which may have been responsible for the positive effect, because music soothes the mind, distracts from tinnitus, and motivates patients to undergo treatment, resulting in a significant impact [19,20,28,29]. Zentones consist of fractal sounds and exert a relaxing effect on the cerebral cortex [19]. Moreover, the number of sound types is expected to increase in the future as the functionality of tinnitus treatment devices and their ability to work with smartphones and other peripheral devices evolve [30]. This suggests that the differences in tinnitus treatment effects, owing to different types of sounds, should be considered in future studies.

We found almost no significant differences among the models in VAS scores regarding distress levels. Although a previous study reported a correlation between the THI and VAS [31], their methods of describing the THI and VAS differed [32]. Moreover, the VAS score was measured by marking the extent to which tinnitus affected the patient. Therefore, we assumed that there would be no difference between the THI and VAS because the VAS is not as specific a measure as the THI in terms of the treatment effect. Additionally, we provided educational counselling during outpatient aural rehabilitation and believe that, after a year or more, the patients became “accustomed to tinnitus”, became accustomed to living with it, and were able to accept it [33]. Moreover, given the lack of differences between the models for the improvement in the VAS, it was possible that our educational counselling was effective for the VAS scores.

The tinnitus distress level is associated with anxiety [34]; therefore, we hypothesized that reducing the tinnitus distress level would also improve the state and trait anxiety. Our results showed the amelioration of the STAI—state and STAI—trait scores for HAs, but not SG HAs or SGs at 1 year. In addition, no significant differences were seen among the models and the CNT at the 2-year follow-up. Our results were consistent with previous studies that noted the HA effects on emotional skills and mental health [35,36]. Moreover, although we expected acoustic therapy to alleviate anxiety as well as cognitive behavior [37], we found that SG HAs and SGs did not improve the STAI scores. Thus, acoustic therapy seemed to ameliorate tinnitus via a different mechanism than HA use and anxiety.

Finally, improvements in the EQS did not significantly differ among the models. The EQS indicates the patient’s response to stress in terms of self, interpersonal, and situational responses. Previous studies, other than those on tinnitus, have reported that treatment interventions improve emotional intelligence [38,39], thereby suggesting that acoustic therapy using SG HAs, HAs, or SGs can cause behavioral changes. Based on this observation, we initially speculated that the ability of individuals to cope with themselves, others, and their surroundings would improve. However, our results suggested that the educational counselling provided to all the patients in our hearing rehabilitation outpatient clinic played an important role, rather than the impacts of different models.

Nevertheless, this study had some limitations. First, the study lacked data on hearing- and pitch-matching tests because TRT was introduced for patients with subjective hearing loss. Therefore, future studies should examine the differences in treatment efficacy according to the degree of hearing loss. Second, although this study demonstrated differences among the models, the mechanism is only speculative and requires further verification using imaging tests, such as fMRI and MEG, or other measuring tools, such as tinnitus-matching or real estimation methods. Finally, although the treatment course was followed for 2 years, further long-term follow-up is required to determine when TRT should be discontinued.

## 5. Conclusions

Our results indicate that TRT using SG HAs, particularly ZEN, and SGs is effective in treating chronic tinnitus, as HAs tend to exacerbate tinnitus with long-term use. In other words, both HAs and SG HAs should be introduced in the early stages of treatment to reduce distress, followed by continued educational counselling for effective tinnitus control. As the functionalities of tinnitus treatment devices and their compatibility with smartphones and other peripheral devices evolve, patients will have increased freedom in selecting and adjusting treatment sounds. Moreover, differences in tinnitus treatment effects based on sound types should be considered in the future.

## Figures and Tables

**Table 1:** Demographic information.

Group	CNT	HA	SG HA		SG		
Subgroup	CNT	HA	ZEN	TTHA/TTC	TT/TTSG	TCI	*p*-Value
Manufacturer	-	Various	Widex	Makitie	Makitie	Sivantos	
Characteristics for SG			ZEN Tone	White Noise, etc.			
**N**	53	28	214	48	31	43	
**Age (years) (mean ± SD)**	56.8 ± 13.4	65.9 ± 10.4	63.2 ± 13.0	62.4 ± 12.4	60.0 ± 12.8	60.2 ± 11.4	ns ^a^
**Sex (male/female)**	22:31	14:14	91:123	25:23	19:12	26:17	ns ^b^

^a^: One-way ANOVA, post hoc Tukey; ^b^: Fisher’s exact test; ns: not significant; SD: standard deviation; CNT: control group; HA: hearing aid; SG: sound generator..

**Table 2:** THI scores based on each device.

Group		CNT	HA	SG HA		SG	
Subgroup		CNT	HA	ZEN	TTHA/TTC	TT/TTSG	TCI
Manufacturer		-	Various	Widex	Makitie	Makitie	Sivantos
Characteristics for SG				ZEN Tone	White Noise, etc.		
**THI**	First visit	30.7 ±19.4	55.4 ± 21.6	45.6 ± 29.0	46.7 ± 27.5	41.0 ± 27.3	38.1 ± 23.5
	1 year	41.1 ± 28.3	36.7 ± 21.3	37.3 ± 24.3	45.4 ± 23.9	41.7 ± 26.8	33.1 ± 23.3
	2 years	35.9 ± 20.9	51.3 ± 19.2	32.3 ± 24.6	43.0 ± 24.2	40.4 ± 25.4	31.8 ± 26.2

CNT, control group; HA, hearing aid; SG, sound generator; THI, tinnitus handicap inventory.

## Data Availability

All the data in this manuscript are available upon request.

## Supplementary Materials

The following supporting information can be downloaded at: https://www.mdpi.com/article/10.3390/audiolres14040057/s1, Figure S1: Group comparison of tinnitus improvement rates.
