# Supplementary material for: Optimizing Tinnitus Management: The Important Role of Hearing Aids with Sound Generators
Source: Audiol Res. 2024 Aug 6;14(4):674–83. doi: 10.3390/audiolres14040057 (PMC11351871; doi:10.3390/audiolres14040057)
Supplement: Supplementary file 1 [file audiolres-14-00057-s001.zip › audiolres-3055053-supplementary.pdf]

Supplementary information

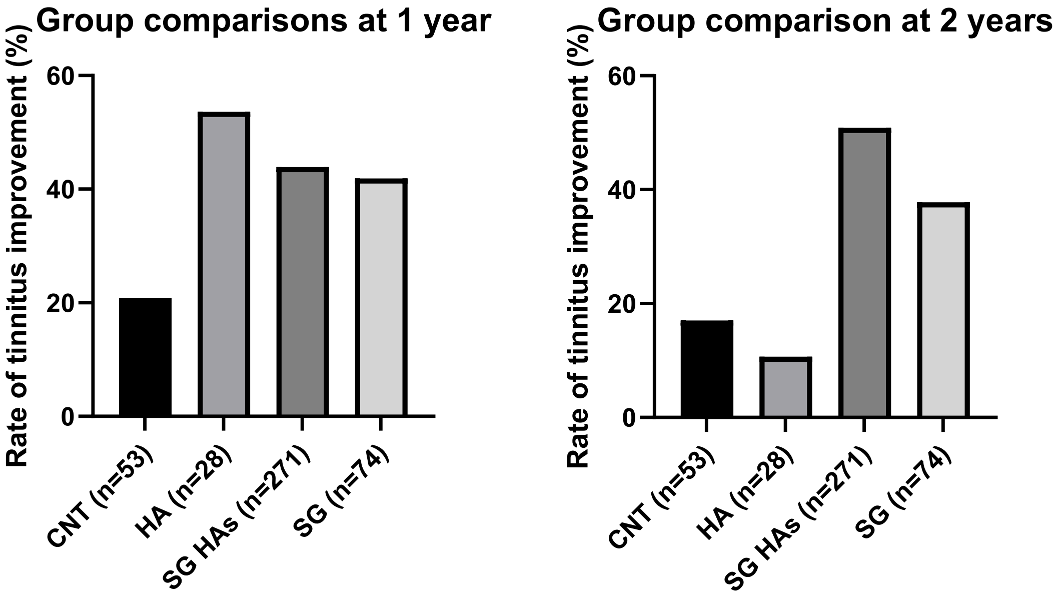

Supplementary Figure S1. Group comparison of tinnitus improvement rates. CNT, control group; HA, hearing aid; SG, sound generators
